# Synergistic Effect of Metal Oxide Nanoparticles on Cell Viability and Activation of MAP Kinases and NFκB

**DOI:** 10.3390/ijms19010246

**Published:** 2018-01-15

**Authors:** Ángela Dávila-Grana, Lara Diego-González, África González-Fernández, Rosana Simón-Vázquez

**Affiliations:** Inmunología, Centro de Investigaciones Biomédicas (CINBIO), Centro Singular de Investigación de Galicia, Instituto de Investigación Sanitaria Galicia Sur (IIS-GS), Universidade de Vigo, Campus Universitario de Vigo, 36310 Pontevedra, Spain; angeladavila17@gmail.com (Á.D.-G.); ldiego@uvigo.es (L.D.-G.); africa@uvigo.es (Á.G.-F.)

**Keywords:** nanotoxicity, ion release, accidental exposure, Np combinations, viability, cell activation, ERK, p38, SAPK/JNK, IκBα

## Abstract

In recent years, there has been an increase in the production of several types of nanoparticles (Nps) for different purposes. Several studies have been performed to analyse the toxicity induced by some of these individual Nps, but data are scarce on the potential hazards or beneficial effects induced by a range of nanomaterials in the same environment. The purpose of the study described here was to evaluate the toxicological effects induced by in vitro exposure of human cells to ZnO Nps in combination with different concentrations of other metal oxide Nps (Al_2_O_3_, CeO_2_, TiO_2_ and Y_2_O_3_). The results indicate that the presence of these Nps has synergistic or antagonistic effects on the cell death induced by ZnO Nps, with a quite marked beneficial effect observed when high concentrations of Nps were tested. Moreover, analysis by Western blot of the main components of the intracellular activation routes (MAPKs and NFκB) again showed that the presence of other Nps can affect cell activation. In conclusion, the presence of several Nps in the same environment modifies the functional activity of one individual Np. Further studies are required in order to elucidate the effects induced by combinations of nanomaterials.

## 1. Introduction

The unusual properties of nanoparticles (Nps) are on the basis of their great expansion due to their potential applications in different areas (energy, food, health, clothing, cars, etc.). This situation has led to an increase in the release of different nanomaterials into the environment and their potential effects on the ecosystem and human health are becoming a major concern [1]. As a consequence, there is a pressing need to fully characterize the toxicological effects that Nps could have when they are released into the environment or come into contact with the human body.

Metal oxide Nps are used in a considerable number of applications. For example, zinc oxide (ZnO) and titanium oxide (TiO_2_) Nps are used in the production of sunscreens and cosmetics due to their capacity to block both UV-A and UV-B rays [2]. This is a potentially important source of Np contamination due to wash-off from individuals into the environment, which can contaminate water and soil. TiO_2_ Nps are also useful for the treatment of waste water [3]. ZnO Nps are present in several chemical sensors and solar cells [4]. Cerium oxide (CeO_2_) Nps can act as oxygen sensors and fuel additives to improve the efficiency of combustion [5] and they can also reduce oxidative stress in biological systems as a free radical scavenger [6]. Other metal oxide Nps include aluminium oxide (Al_2_O_3_) and yttrium oxide (Y_2_O_3_) Nps. The former are used in personal care products as well as in a wide range of applications in industry [7] such as abrasive agents, wear-resistant coatings or as additives for polymeric nanomaterials designed for drug delivery and thermal agents [8]. Y_2_O_3_ Nps are widely used for various rare earth-doped materials and they also play an important role in potential applications in biological imaging and photodynamic therapy [9,10].

Due to the diverse range of applications of these metal oxide NPs, and also to the number of companies and research groups working with them, the possibility that some of these Nps could enter the human body (either by inhalation, oral ingestion, dermal penetration or injection) has increased significantly in recent years. Furthermore, many workers and academic researchers may be exposed to Nps during their production and manipulation [11].

To date, toxicity studies on Nps have mostly been focused on individual Nps and not on the combination of several such species [12]. However, given the rapid development of this technology, it is expected that the amount of Nps released into the environment will increase in the near future. This raises the possibility that various types of Nps could be found in the same medium (water, air, ground, food, organisms) and the effect of some Nps could be potentiated, inhibited or remain unaffected by the presence of other nanomaterials. Nevertheless, investigations into the potential hazards of physical mixtures of Nps are limited [13,14]. This lack of toxicological data on nanomaterial combinations makes it difficult to determine if there is any risk associated with exposure to combined nanomaterials. Thus, there is an urgent need to evaluate the effects caused by combinations of Nps.

In recent years, we have studied the cell toxicity induced by several metal oxide NPs in human cell lines [15,16] and microalgae [17], and have also analysed the potential hazardous effects on immune cells. Our results indicate that some commercial Nps tested in our laboratory, namely Al_2_O_3_, CeO_2_, TiO_2_ and Y_2_O_3_ Nps, do not induce toxicity in human cell lines, although some effects were observed for Y_2_O_3_ Nps on microalgal cell growth [17]. In contrast, ZnO Nps showed toxicity in a dose-dependent manner in all cell lines and marine microorganisms tested.

Our results with ZnO Nps are consistent with those obtained by other authors in that they showed toxicity on bacteria [18], peripheral blood mononuclear cells (PBMCs) [19] or different cell lines [20,21], and this effect could be attributed to the release of dissolved zinc ions into the medium [22,23].

Preliminary tests conducted in our laboratory on Jurkat cells proved that ZnO (50 and 100 µg/mL) causes the activation of three mitogen-activated protein kinases (MAPKs), thus inducing the phosphorylation of p38, ERK 1/2 and SAPK/JNK, together with the degradation of IκBα, the NFκB inhibitor. In contrast, Al_2_O_3_, CeO_2_ and TiO_2_ only showed activation of some of these routes and the degradation of IκBα at high concentrations (100 µg/mL) [24]. Moreover, Jurkat cells were more sensitive to activation of these signalling proteins when compared to NCI-H460 cells [25]. Other studies have been carried out to evaluate the cellular toxicity induced by some metal oxide Nps in different cell types and the activation of the aforementioned pathways has been highlighted [26,27,28,29,30]. For instance, the role of p38 has also been described in the apoptosis induced by ZnO Nps in human fibroblasts [26]. In contrast to Jurkat cells, where only ERK1/2 and p38 were activated by CeO_2_ Nps mainly after pre-stimulation of the cells, in human hepatoma cells the SAPK/JNK was also activated by these Nps and activation of the three MAPKs was associated with oxidative stress and reduced cell viability at concentrations ≥50 µg/mL [27]. Interestingly, TiO_2_ Nps induced activation of ERK1/2 and p38 in a human neutrophil [29] and a bronchial epithelial cell line [30] as described for Jurkat cells [24]. However, the activation of these signalling proteins was associated with the inhibition of apoptosis in the lymphocytic and neutrophil cell line, while in the bronchial epithelial cells the Nps induced apoptosis and oxidative stress. Moreover, the activation of both MAPKs was also associated with increased IL-8 levels in the neutrophils and bronchial epithelial cells but decreased IL-8 gene expression in the lymphocytic cell line.

Bearing the above information in mind, we decided to evaluate whether the activation routes involving the MAPKs and the NFκB factor were altered when ZnO Nps were present in combination with other Nps.

MAPKs are serine-threonine kinases that are activated by extracellular stimuli and mediate intracellular signalling associated with a variety of cellular activities including cell proliferation, differentiation, survival, death, and transformation [31]. MAPKs are part of a system composed of three sequentially activated kinases and, like their substrates, MAPKs are regulated by phosphorylation [32]. There are three relevant MAPK subfamilies: extracellular signal-regulated kinase (ERK), the p38 and the c-Jun amino-terminal kinase (JNK), which is also called stress-activated protein kinase (SAPK) [32]. The ERK pathway has long been associated with cell growth, cell proliferation and cell survival. In contrast, the activation of the p38 pathway is required for apoptosis induction. SAPK/JNK activity can mediate apoptosis, proliferation, or survival, depending on the stimuli and cellular conditions [33].

NFκB (nuclear factor kappa-light-chain-enhancer of activated B cells) is a complex protein that controls DNA transcription. Its activity is regulated by the cytoplasmic degradation of its inhibitory unit, the IκBα. In response to a variety of stimuli, IκBα is rapidly phosphorylated and degraded by proteasomes that release the NFκB transcription factor. This protein is translocated to the nucleus and modulates the expression of a number of genes [34]. Thus, the level of degradation of the IκBα inhibitor can be used to study the activation of the NFκB pathway [35].

The objective of the study reported here was to understand the interactions between several metal oxide Nps (Al_2_O_3_, CeO_2_, TiO_2_ and Y_2_O_3_) in combination with ZnO Nps on cell viability and the effect induced by these combinations on the activation of the MAPKs and the NFκB factor.

The concentrations tested were in the range from low/moderate to very high in order to obtain information about the dose-dependence and potential effect induced by the nanomaterials after transient exposure or chronic/bioaccumulation (where the local concentration reached might be very high). Moreover, extremely high concentrations were also used to assess whether the effects observed at any concentration were dose-dependent or if paradoxical effects could be observed.

## 2. Results

### 2.1. Synergistic Effect of Nanoparticles on Cell Viability

A cell viability assay (MTS) was performed to assess the potential synergistic effects of different metal oxide Nps on the cytotoxicity induced by ZnO Nps on Jurkat cells. The results are expressed as mean ± SD (standard deviation) of three independent experiments (*n* = 3). The IC50 of the ZnO Nps in Jurkat cells was about 60 µg/mL [24] and, as a consequence, this concentration was used in combination with increasing concentrations of other metal oxide Nps.

**Figure 1:**
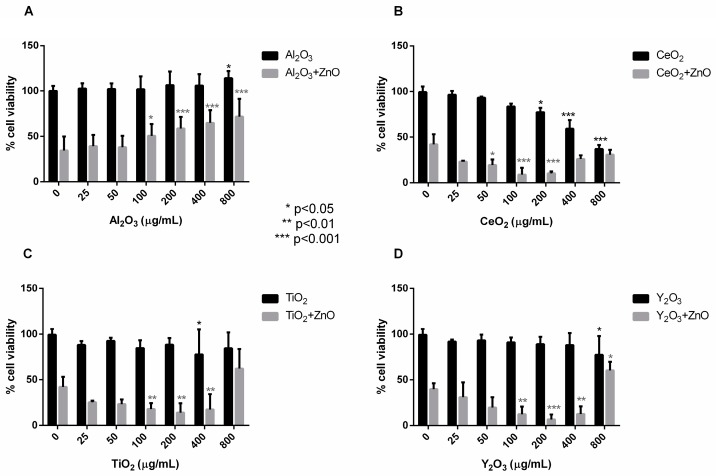
Synergisitc effect on Jurkat cell viability induced by the combination of ZnO with Al_2_O_3_ (**A**), CeO_2_ (**B**), TiO_2_ (**C**) and Y_2_O_3_ Nps (**D**). An increase in cell viability was determined by the colorimetric assay Cell Titer 96^®^ AQueous One Solution Cell Proliferation Assay for 100–800 µg/mL Al_2_O_3_ Nps combined with 60 µg/mL of ZnO and a decrease in viability for the combination of ZnO Nps with CeO_2_, TiO_2_ and Y_2_O_3_ Nps (*n* = 3). In the statistical analysis, the viability of the individual metal oxide Nps at different concentrations were compared to control cells (untreated) and the viability of the metal oxide Np combinations were compared to the viability of ZnO Nps alone. Statistically significant differences are marked with asterisks.

Of all the Nps tested, Al_2_O_3_ Nps were the only ones that did not affect cell survival at concentrations from 25 µg/mL to 800 µg/mL (Figure 1A). These Nps even induced a small increase in the cell viability at the highest concentrations tested. The combination of different concentrations of Al_2_O_3_ Nps with 60 µg/mL ZnO Nps led to an increase in the viability of the Jurkat cells compared to ZnO Nps alone. This increase in the cell viability was dose-dependent (Figure 1A) and the combination with 800 µg/mL of Al_2_O_3_ Nps increased the viability (by a factor of two compared with ZnO Nps alone). Thus, Al_2_O_3_ Nps seem to have a protective effect on cells when they are in contact with ZnO Nps.

In contrast to the above, CeO_2_ and TiO_2_ Nps affected the viability in a dose-dependent manner, although in both cases the IC50 was higher than 400 µg/mL (Figure 1B,C). In the case of Y_2_O_3_ Nps, only at the highest concentration tested, there was a decrease in the detected viability, but in any case, the viability was never less than 75% (Figure 1D).

The combination of CeO_2_, TiO_2_ and Y_2_O_3_ Nps with 60 µg/mL ZnO Nps had a stimulatory effect on the toxicity towards the cells and this is reflected by a decrease in cell viability with respect to that observed with ZnO Nps alone (Figure 1). The viability decreased in a dose-dependent manner except for Y_2_O_3_ at 800 µg/mL. Interestingly, in this case, the viability of the combined Nps was around 20% higher than that of the cells in the presence of 60 µg/mL ZnO Nps alone, and about 50% higher than the viability induced by the mixture with 400 µg/mL Y_2_O_3_ Nps.

A similar trend was observed for CeO_2_ and TiO_2_ Nps at the highest concentrations, although the SD in this case was quite high. Hence, the CeO_2_, TiO_2_ and Y_2_O_3_ Nps had a synergistic effect on the toxicity caused by ZnO Nps towards Jurkat cells except at very high concentrations, in which case they had a protective effect on the viability of the cells.

Two approaches were considered in an effort to eliminate the possible interference of Nps in the viability colorimetric method (in which absorbance is measured). The first approach was to analyse the signal given by Nps alone at high concentration and to subtract this from the absorbance of the cells incubated with the Nps. The second approach was to use a different non-colorimetric method based on real-time impedance. The impedance measurement requires the cell to grow attached to the electrode and, for this reason, the adherent macrophage-differentiated cell line (THP1) was used rather than Jurkat cells. An increase in the cell viability was detected for THP-1 cells incubated with ZnO Nps at 60 µg/mL and a high concentration of another metal oxide Np (Figure S1), mainly with CeO_2_ and TiO_2_ Nps. Interestingly, the Al_2_O_3_ Nps at 800 µg/mL were toxic on THP1 cells and the combination with ZnO Nps also increased the cytotoxicity.

### 2.2. Solubility of the ZnO Nanoparticles in the Nanoparticle Mixtures

Ion release and the subsequent loss of homeostasis has been reported to be one of the main mechanisms of toxicity associated with ZnO Nps [22,23]. As a result, the total amount of Zn^2+^ released from the ZnO Nps, either alone or in the presence of other metal oxide Nps, was measured in the supernatant after 24 h of incubation with the Jurkat cell line.

The same ZnO Np concentration as used in the viability experiments (60 µg/mL), with the addition of Al_2_O_3_, CeO_2_, TiO_2_ and Y_2_O_3_ Nps at low (50 µg/mL) and high (800 µg/mL) concentrations, was incubated with the Jurkat cells and the Zn^2+^ ions released into the culture medium were quantified by ICP-OES.

**Figure 2:**
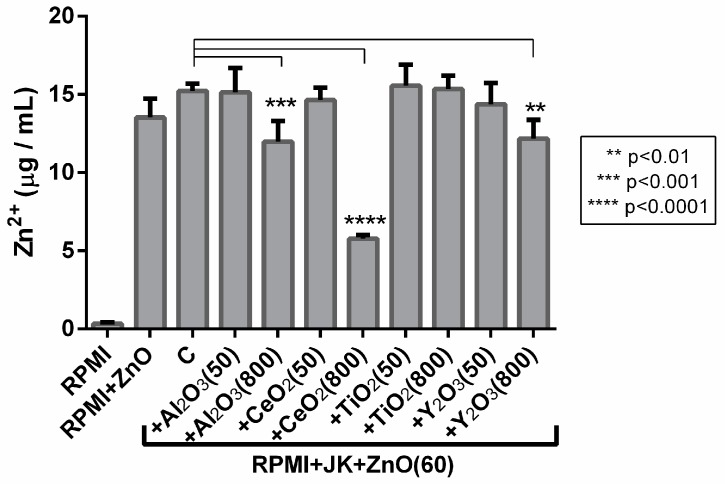
Concentration of dissolved Zn^2+^ by ICP-OES. The concentration of Zn^2+^ in a culture medium released by 60 µg/mL ZnO Nps is represented. In all cases the concentration of Zn^2+^ is the same or higher when ZnO Nps are combined with metal oxide Nps at 50 µg/mL and lower when combined with metal oxide Nps at 800 µg/mL, except for TiO_2_ Nps (*n* = 2). C: control sample, ZnO Nps at 60 µg/mL in the presence of Jurkat cells incubated in RPMI. The metal oxide Np combinations were compared to this sample for the statistical analysis. Asterisks and lines highlight the statistically significant differences.

The levels of Zn^2+^ ions released from ZnO Nps and combinations of Nps after their incubation with the cells are represented in Figure 2 along with the result for ZnO Nps alone in the culture medium (RPMI). Furthermore, the culture medium (free of Nps) was also tested and a low Zn^2+^ ion content of about 0.3 µg/mL was found.

The amount of Zn^2+^ released from ZnO Nps in the culture medium was about 13.5 µg/mL and after incubation with the Jurkat cells this increased to 15.2 µg/mL. In the case of ZnO Nps combined with the metal oxide Nps at 50 µg/mL, the amount of dissolved Zn^2+^ was similar in all supernatants (Figure 2).

Interestingly, when the concentration of the metal oxide Nps was increased up to 800 µg/mL in the Np mixtures, the amount of free Zn^2+^ ions decreased significantly for all the Nps tested except for TiO_2_ Nps. Moreover, the lowest amount of dissolved Zn^2+^ was obtained with the combination of ZnO Nps and CeO_2_ Nps (Figure 2). Thus, the metal oxide Nps at high concentration may inhibit the release of Zn^2+^ ions from the ZnO Nps when they are mixed, probably by inducing their precipitation and decreasing their solubility [36].

### 2.3. The Effect of Combined Metal Oxide Nps on Activation of MAPKs and NFκB Factor

The phosphorylation pattern of MAPKs and the activation of NFκB in Jurkat cells incubated with the individual or mixed Nps was tested by Western blot and the bands were quantified with specialized software that measures pixel intensity. The ZnO Nps were added at 5 µg/mL and the metal oxide Nps were tested at 5 and 50 µg/mL to study the potential dose-dependent effect on the regulation of activation. GAPDH was used as a loading control with all the MAPKs.

**Figure 3:**
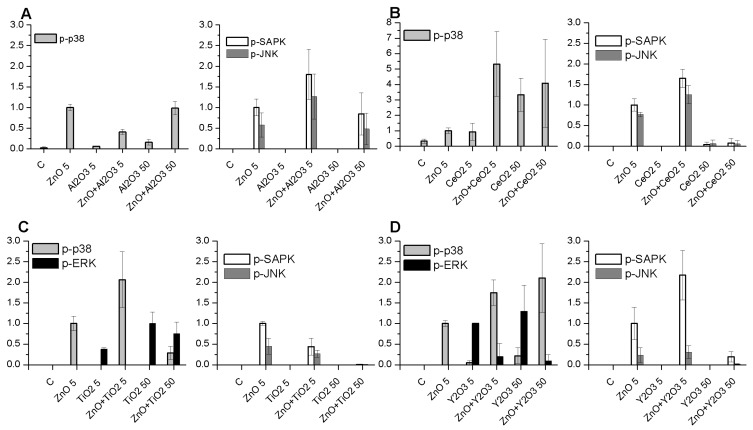
Expression of p-p38, p-ERK and p-SAPK/JNK in Jurkat cells. The graphs show the expression of p-p38, p-ERK and p-SAPK/JNK in cells treated with Al_2_O_3_, CeO_2_, TiO_2_ and Y_2_O_3_ (**A**–**D**) at different concentrations (5 and 50 µg/mL) and combined with 5 µg/mL ZnO.

The results of the Western blot experiments showed that the p-p38 pathway was activated by Al_2_O_3_ and Y_2_O_3_ Nps at 50 µg/mL and CeO_2_ Nps at both concentrations tested, whereas Al_2_O_3_ and Y_2_O_3_ at 5 µg/mL and TiO_2_ Nps showed very weak or no activation (Figure 3 and Figure S2) at both concentrations.

Moreover, activation of p-p38 decreased when ZnO Nps were in the presence of Al_2_O_3_ Nps compared with the activation found with ZnO Nps alone, but it was higher than the activation induced by the individual Nps except for TiO_2_ Nps at 50 µg/mL, where the band was very weak. In contrast, the combination of CeO_2_ and Y_2_O_3_ Nps with ZnO Nps increased the phosphorylation of the p38 protein, and in the case of CeO_2_ Nps the highest signal of all the combinations tested was obtained.

Regarding p-ERK (Figure 3 and Figure S2), activation of this route was not induced by either Al_2_O_3_ or CeO_2_ Nps and nor did the combination with ZnO Nps cause activation. However, TiO_2_ and Y_2_O_3_ Nps alone activated p-ERK at both concentrations, but when mixed with ZnO Nps the intensity of the bands corresponding to the phosphorylated protein decreased or was not detectable.

Activation of p-SAPK/JNK (Figure 3 and Figure S2) was not induced by any of the metal oxide Nps (Al_2_O_3_, CeO_2_, TiO_2_ and Y_2_O_3_) at the concentrations tested, except for ZnO Nps, which showed a high level of activation at 5 µg/mL. The combination of ZnO Nps with low doses of Al_2_O_3_, CeO_2_ and Y_2_O_3_ Nps increased the phosphorylation, but the opposite result was observed for TiO_2_ Nps. When the Np concentration increased up to 50 µg/mL, the intensity of the bands decreased in all cases compared with those corresponding to the ZnO Nps alone. It is worth noting that for CeO_2_ and TiO_2_ Nps, the bands corresponding to the phosphorylated protein were very weak or were not detectable.

**Figure 4:**
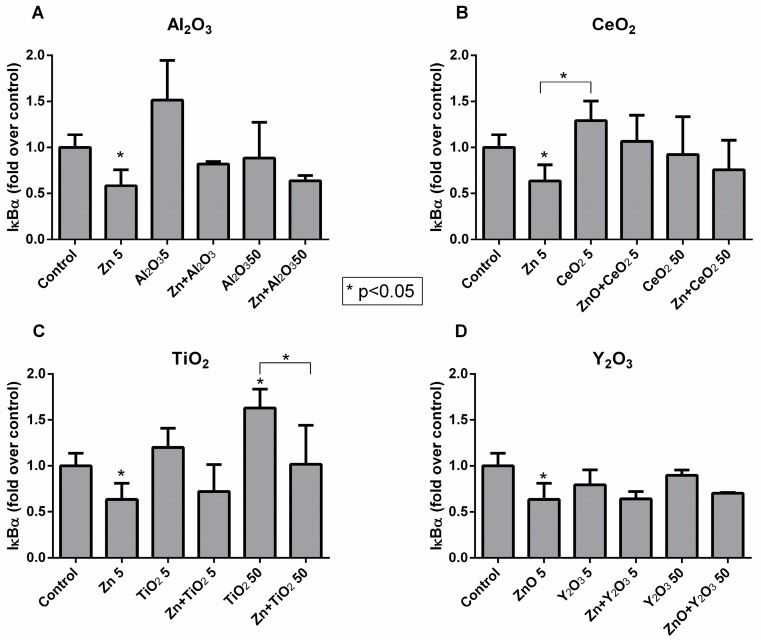
Expression of IκBα in Jurkat cells. The graphics show the expression of IκBα in cells treated with Al_2_O_3_ (**A**), CeO_2_ (**B**), TiO_2_ (**C**), Y_2_O_3_ (**D**) Nps at different concentrations (5 and 50 µg/mL) and combined with 5 µg/mL ZnO Nps for 3 h. Asterisks are used to indicate statistically significant differences between treated cells and untreated cells (control). Lines and asterisks mark differences between treated cells.

The activation of the NFκB pathway is characterized by the degradation of the IκBα inhibitor. This degradation was quantified by measuring the intensity of the IκBα band in the membranes with cell lysates incubated with the Nps and comparing the results with those obtained with untreated cells (Figure 4A–D).

The results show that all metal oxide Nps at 5 µg/mL increased the IκBα protein level compared to the control cells, except for Y_2_O_3_ and ZnO Nps. In fact, ZnO Nps induced a significant degradation of the protein and hence activation of the NFκB pathway. However, only TiO_2_ Nps at a higher concentration (50 µg/mL) led to an increase in the IκBα protein level, whereas the rest of the metal oxide Nps induced a small decrease or did not cause a change.

Overall, the combination of the metal oxide Nps with ZnO Nps increased the IκBα protein level compared to that found with ZnO Nps alone.

## 3. Discussion

The potential risks related to the release of nanomaterials into the environment and subsequent effects on the ecosystem and human health are increasing concerns. Although there is abundant bibliography on the toxicity caused by individual metal oxide Nps, the possibility of finding different Nps in the same medium should be considered and, hence, potential synergistic/antagonistic effects should be evaluated. However, detailed information on the toxicity of combinations of different metal oxide Nps has not been published to date. As a consequence, the focus of this study was the characterization of the toxicity and cell activation caused by ZnO Nps in combination with other metal nanoparticles such as Al_2_O_3_, CeO_2_, TiO_2_ or Y_2_O_3_.

ZnO Nps have proven to be toxic for several cell types [15,16] and the IC50 was approximately 60 µg/mL in the Jurkat lymphocytic cell line [24].

Accidental exposure to Nps may occur via oral, dermal or respiratory tract routes and, therefore, many cell types can be exposed. NPs could also reach the blood or lymph and encounter several cells like lymphocytes or macrophages.

All of the metal oxide Nps studied, besides ZnO Nps, caused toxicity at high concentration except for Al_2_O_3_ Nps. CeO_2_ and TiO_2_ Nps decreased the cell viability at concentrations above 400 µg/mL, and with Y_2_O_3_ Nps the cells only showed toxicity at the highest concentration tested (800 µg/mL). When these metal oxide Nps were combined with ZnO Nps (at 60 µg/mL) and incubated with Jurkat cells, the cell viability was modified compared to incubation with the individual Nps. Al_2_O_3_ Nps were the only Nps that had a protective effect in combination with ZnO Nps. This combination reduced the toxicity exerted by these Nps in a dose-dependent manner.

In contrast to the above, the combination of ZnO Nps with CeO_2_, TiO_2_ and Y_2_O_3_ Nps had a synergistic effect on the toxicity caused by the ZnO Nps on Jurkat cells. This effect was also dose-dependent and, interestingly, it was inverted at the highest concentrations employed (400–800 µg/mL). This protective effect on the cell viability caused by Nps at high concentration was confirmed in macrophage-differentiated THP-1 cells by an impedance-based viability assay (supplementary information).

The toxic effect caused by Nps in cells may be explained by the release of Zn^2+^ ions into the culture medium. ZnO Nps are partially soluble and an increase in their solubility could be related to the effect that these Nps have on cells. In fact, the matrix effect on the solubility of ZnO Nps has been described previously [37,38]. For instance, the presence of albumin increases the solubility of Nps. In our work, the presence of serum and cells could also increase the Np solubility and, for this reason, the concentrations found were high. However, the solubility of ZnO Nps did not increase when they were combined with CeO_2_, TiO_2_ and Y_2_O_3_ Nps (at low to moderate concentration), although they did induce high cell toxicity. Moreover, the increased cell viability in the cells incubated with ZnO Nps in the presence of Al_2_O_3_ NPs was not related to a significant change in the ZnO Np solubility.

These results suggest additive/synergistic effects induced by the Nps that are independent of Zn^2+^ ion release.

In contrast to the situation described above, in all combinations of ZnO with the other metal oxide Nps, a decrease in cell toxicity was observed when metal oxide Nps were at the highest concentrations (400 or 800 µg/mL). This could be due to the fact that when ZnO Nps are combined with metal oxide Nps at 800 µg/mL the amount of Zn^2+^ ions dissolved in the medium is lower than for the ZnO Nps alone. In fact, statistically significant differences in the amount of free Zn^2+^ ions in the absence or presence of other Nps were found in all cases apart from TiO_2_ Nps. Thus, although the release of Zn^2+^ ions from ZnO Nps to the medium could partially explain the cytotoxicity caused by these Nps, it might not be the only mechanism involved in the toxicity. In fact, although TiO_2_ Nps induced the lowest inhibition of ion release at the highest concentration tested, they also led to a relevant increase in cell viability in combination with ZnO Nps. This result also proves that the recovery of the cell viability was not due to a physical phenomenon such as aggregation of the ZnO Nps due to a high Np concentration in the medium, but rather a physiological issue related to synergy between different Nps.

These results could have several explanations. One is that the highest concentration of Nps could affect the colorimetric assay of MTS, although the background given by Nps alone was always low and constant for all concentrations tested. However, this possibility was ruled out due to the observation of the same phenomenon in THP-1 cells in a non-colorimetric assay (supplementary information).

Another, and more feasible, possibility is that a high Np concentration leads to the precipitation of the ZnO Nps and, thus, decreases the solubility [36]. It is important to take into account that although ZnO Nps could precipitate to a greater extent, the combination of ZnO with other metal oxide Nps at high concentrations decreased the size of the Np physical mixtures when compared with Nps at a low concentration, as seen by DLS in water (Table S1).

In order to confirm that the combination of Nps was responsible for inducing different effects on the cells when compared with the individual Nps, we characterized how these Np combinations altered the MAPK signalling and the NFκB factor pathway.

The MAPK family participates in the regulation of growth, survival, adaptation and apoptosis of cells in response to a stimulus. The ability of Nps to interact with these signalling pathways could partially explain their cytotoxicity. Three groups of MAPK signalling proteins were investigated, i.e., ERK, p38 and SAPK/JNK, in association with the NFκB factor.

Previous experiments performed in our laboratory showed a strong signal for the activated MAPKs and NFκB when cells were treated with ZnO Nps at 50 µg/mL [24,25]. In this work, however, we tried to find the appropriate concentration of ZnO Nps that could induce a moderate signal that would allow the effect of Np combinations to be analysed, because a very high ZnO Np signal alone would not allow the detection of small modulations at the protein level in the presence of other Nps. For this reason, 5 µg/mL of ZnO was used to study the effect of combined Nps on Jurkat cells. At this Np concentration, the p38, SAPK/JNK and NFκB (Figure 4) are activated [24].

The effect of the individual and combined Nps on Jurkat cells are summarized in Table 1. TiO_2_ Nps did not activate any of the MAPKs studied except for ERK at 50 µg/mL. CeO_2_ and Al_2_O_3_ Nps activated p38 only and Y_2_O_3_ Nps activated the ERK signalling protein at both concentrations tested and p38 at 50 µg/mL. The combination with ZnO Nps induced changes in almost all of the phosphorylated proteins and this led to a significant increase in the protein level, such as the case of p-p38 and Y_2_O_3_ Nps, or even a lack of activation such as the expression of p-SAP/JNK by ZnO Nps in the presence of CeO_2_ or TiO_2_ Nps.

Concerning the NFκB factor, all of the metal oxide Nps caused minor changes in the level of the inhibitory IκBα protein, with the exception of ZnO Nps at 5 µg/mL and TiO_2_ Nps at 50 µg/mL, which induced a significant decrease and increase, respectively. Both changes in the protein level could be related with NFκB activation [35]. In general, the combination of all of the metal oxide Nps with the ZnO Nps reduced the degradation of the IκBα inhibitor induced by ZnO Nps alone, but increased the degradation induced by the individual metal oxide Nps. In summary, significant changes in the inhibitory protein were not detected for the Np combinations when compared to the untreated cells.

Some of the effects observed could be additive rather than synergistic effects. However, in both cases the result expected would be a decrease in cell viability together with an increase in cell activation, and hence a different outcome from the effect induced by the individual Np. Furthermore, the decrease in the cell viability induced by the mixture of the metal oxide Nps with ZnO Nps was dose-dependent at low or moderate concentration, but completely inverted when high concentrations were used. Therefore, a mixture of additive/synergistic/antagonistic effects occurred depending on the concentration used.

While Al_2_O_3_ Nps were the only Nps that showed a clear protective effect in combination with ZnO Nps in Jurkat cells, TiO_2_ Nps only protected the cells from MAPK and NFκB activation but not from the cytotoxic effect at concentrations <400 µg/mL. This finding is consistent with the results observed in human nasal mucosa, in which TiO_2_ Nps were able to reduce the genotoxic effects induced by ZnO Nps, although changes in the cytotoxicity results were not found for the physical mixture of both Nps at a low concentration [38]. On the other hand, the combination of ZnO Nps with CeO_2_ and Y_2_O_3_ Nps also increased the toxicity at ≤400 µg/mL and the activation of p-p38 in a clear synergistic effect. The increased cell toxicity observed with CeO_2_ Nps in combination with ZnO Nps at a low to moderate concentration, possibly mediated by the activation of p38 in the cells, was also described in the bacterium *Nitrosomonas europaea* [39]. The increased toxicity in the bacteria was also independent of the ZnO solubility, a finding that is consistent with our results. Moreover, the authors characterized the combination of TiO_2_ and CeO_2_ Nps and identified a protective effect of TiO_2_ Nps on the toxicity caused by CeO_2_ Nps [39].

Apart from some scarce studies already available in the literature, there is very little information on the potential synergistic effect of Nps of different natures. Our study contributes to the knowledge of the modified toxicity of ZnO Nps in combination with other metal oxide Nps with different metal compositions. The results show how the dose-effect induced by Np mixtures could be different from that one observed for individual Nps, Moreover, the toxicity induced by a single type of Np can be even modified (increased or decreased) by the co-exposition with Nps of different compositions. These findings are relevant for occupational risk assessments. Besides genetic and individual susceptibility to a particular nanomaterial, the potential co-exposure with other nanomaterials could be responsible of different toxicological outcomes or even protect the workers from inflammation or immune cell activation, among other risks. Similarly, the co-exposure effect in cells is very much dependent on the dose and, in some particular cases, the biological effect induced by Nps is drastically different at low or moderate concentrations, compared with high concentrations.

## 4. Materials and Methods

### 4.1. Nanoparticles

Metal oxide Nps from different manufacturers and with different sizes were used (Table 2). These Nps were part of the HINAMOX project (7th EU framework program). The physicochemical characterization was carried out in the laboratory of Sergio Moya (CIC biomaGUNE) and the relevant data are shown in Table 2. In culture media, all of the Nps showed a negative Z potential and a better dispersion than in water when they were previously treated with serum [15,16].

A 10 mg/mL suspension of each Np was prepared in milli-Q water (Ultramatic, Wasserlab, Navarra, Spain), previously filtered through a 0.22 µm filter (Fast Flow & Low Binding Millipore, Merck Millipore, Billerica, MA, USA), and 10% (*v*/*v*) fetal bovine serum (FBS) (PAA laboratories, Pasching, Austria) was added. The suspension was sonicated for 10 min in a Branson ultrasound bath (Branson 1510, Danbury, CT, USA) at low frequency (47 KHz) in order to break up the aggregates. The sonicated suspensions were diluted in RPMI 1640 medium (Gibco, Thermo Fisher Scientific, Waltham, MA, USA) supplemented with 10% FBS to the working concentrations. The sterility of Nps was preserved in all cases. For each replicate, fresh suspensions and dilutions of Nps were prepared.

Aqueous suspensions of Np mixtures were characterized by DLS (Malvern Zetasizer Nano ZS) for size and zeta potential. Three measurements were recorded for each sample (Table S1, Supplementary Materials).

### 4.2. Cells

The Jurkat cell line was obtained from ATCC (American Type Culture Collection, Middlesex, UK).

Cells were seeded in a 75 cm^2^ culture flask (Sarstedt, Nümbrecht, Germany) in RPMI 1640 medium supplemented with 10% FBS, heat-inactivated (60 min, 56 °C), 2% Penicillin-Streptomycin (Gibco). Cells were maintained in growth in an environment of humidified air containing 5% CO_2_ and at 37 °C. Cells were subcultured every 2–3 days.

### 4.3. Cell Viability Assay (MTS)

Np dilutions were prepared in 96 well U plates (Tissue Culture Plate, 96 well, U-Bottom, Falcon, Corning, NY, USA) in order to obtain concentrations of 25, 50, 100, 200, 400 and 800 µg/mL of Al_2_O_3_, CeO_2_, TiO_2_ and Y_2_O_3_ in the combination. These Nps were combined with ZnO Nps at the IC50 concentration, 60 µg/mL, and incubated with Jurkat cells. For each Np, three independent experiments were performed.

The cell viability assays were carried out at a density of 6 × 10^4^ Jurkat cells per well in a 96-well plate (Tissue Culture Plate, 96-well, Flat-Bottom, Falcon) and the cells were left in the incubator for 24 h prior to the addition of the Nps. The cells were subsequently incubated with the combination of Nps for a further 24 h.

Once the incubation had finished, a colorimetric cell viability assay was performed using the Cell Titer 96^®^ AQueous One Solution Cell Proliferation Assay kit (Promega, Fitchburg, WI, USA) according to the manufacturer’s instructions.

The supernatant was removed from the plates by centrifugation at 600× *g* for 1 min at 4 °C (Sigma 2-16 KL Sartorius, Göttingen, Germany). The MTS stock solution was then diluted 1:6 (*v*/*v*) in cell culture medium and 120 µL of the diluted solution per well were added. The plates were incubated for 1 h and centrifuged again. Afterwards, 100 µL of the supernatant per well were placed in a clean plate in order to remove possible interferences caused by Nps.

In order to obtain a positive control for cell death, 100 µL of 5% Triton X-100 (Sigma-Aldrich, Steinheim, Germany) were added to some wells 1 h before the addition of the diluted MTS solution. RPMI culture medium and Nps alone were used as negative controls for cells alone and cells with NPs, respectively. Finally, absorbance was measured at 490 nm on an Envision multidetector (Perkin-Elmer Inc., Norwalk, CT, USA). The MTS assay is based on the conversion of a tetrazolium salt [3-(4,5-dimethylthiazol-2-yl)-5-(3-carboxymethoxyphenyl)-2-(4-sulfophenyl)-2*H*-tetrazolium] into a coloured and soluble product in the culture medium, namely formazan. This coloured product results from the mitochondrial activity of living cells. The quantity of formazan produced by dehydrogenase enzymes, measured at 490 nm, is directly proportional to the number of living cells. Cell viability, expressed as a percentage, was calculated as follows:

% viability =([A]_treatment_/[A]_control_) × 100 (1)

[A]_treatment_ is the absorbance of the cells incubated with the Nps minus the absorbance of the Nps, and [A]_control_ is the absorbance of the untreated cells minus the absorbance of the culture medium.

### 4.4. Quantification of the Zn*^2+^* Ions Released from ZnO Nanoparticles

In order to ascertain the concentration that would be available to the cells, the amount of dissolved Zn^2+^ ions was measured by inductively coupled plasma-optical emission spectroscopy (ICP-OES). For this purpose, Np dilutions were prepared in 96 well U plates to obtain combinations of 60 µg/mL ZnO Nps with 50 and 800 µg/mL of Al_2_O_3_, CeO_2_, TiO_2_ and Y_2_O_3_ Nps.

As in the previous experiment, a density of 6 × 10^4^ Jurkat cells per well was seeded and, after 24 h of incubation, the Nps were added; the cells were incubated for a further 24 h. ZnO Nps in culture medium and medium free of Nps were also tested. The plate content was subsequently collected in Eppendorf tubes, which were centrifuged at 13,200 rpm, 5 min, 4 °C and the supernatant was collected in clean tubes. The amount of Zn^2+^ present in the supernatant was determined in two independent experiments in duplicate by ICP-OES at CACTI (University of Vigo, Spain) using a Perkin-Elmer Optima 4300 DV Spectrometer (Waltham, MA, USA) with indium as internal standard.

### 4.5. Cell Extracts

In order to obtain cell extracts, 5 × 10^6^ Jurkat cells were seeded in a 25 cm^2^ flask (Sarstedt, Germany) in RPMI without FBS. After 2 h of incubation, culture medium without FBS, alone or with Nps, was added. Nps were added to the flask at a concentration of 5 or 50 µg/mL for Al_2_O_3_, TiO_2_, CeO_2_ and Y_2_O_3_ Nps, either alone or combined with 5 µg/mL of ZnO Nps. The cells were incubated with the Nps for 3 h and then washed by centrifugation (1200 rpm, 5 min, 4 °C) in a Sorvall ST 16R centrifuge (Thermo Scientific Inc., Waltham, MA, USA) followed by a wash with cold PBS in an Eppendorf 5415 R centrifuge (Eppendorf AG, Hamburg, Germany).

Cell extracts were prepared in a lysis buffer with 10 mM Tris.HCl pH 8, 150 mM NaCl, 2.5 mM EDTA and 1% NP-40 detergent. This buffer was supplemented with 10% protease inhibitor (Complete Mini, Sigma-Aldrich) and 10% phosphatase inhibitor (PhosSTOP, Roche Ltd., Basel, Switzerland). The lysates were centrifuged at 13,200 rpm, 4 °C, for 5 min to remove cellular residues. Three independent experiments were performed for each combination and each Np.

### 4.6. Western Blot

Cell extracts were resolved by SDS-PAGE and then transferred onto a polyvinylidene difluoride (PVDF) (Immun-Blot^®^ 0.2 µm, BioRad Laboratories, Hercules, CA, USA) membrane. The membranes were washed with Tris buffer saline supplemented with 1% Tween 20 (TBST) and then blocked with 5% skimmed milk (Sigma-Aldrich Co., Steinheim, Germany) in TBST with constant agitation for 1 h at room temperature (RT).

The membranes were probed with antibodies to determine the expression levels of protein. Therefore, membranes were incubated with 10 mL of p-p38 rabbit monoclonal antibody 1:5000, p-ERK1/2 1:20,000 or p-SAPK/JNK 1:10,000 (Cell Signaling Technology, Danvers, MA, USA), all diluted with TBST. Anti-GAPDH (Sigma-Aldrich Co., Steinheim, Germany) diluted 1:600,000 was used as a control.

In order to observe the degradation of IκBα, a rabbit monoclonal antibody (Cell Signaling Technology, Danvers, MA, USA) diluted 1:30,000 was used. Goat anti-rabbit IgG polyclonal antibodies conjugated to HRP (Cell Signaling Technology, Danvers, MA, USA) diluted 1:50,000 in TBST with 2.5% skimmed milk were used as secondary antibodies.

Membranes were developed using the ClarityTM Western ECL Substrate kit (BioRad Laboratories) in the ChemiDoc XRS imaging system (BioRad Laboratories), and bands were detected and quantified with Image Lab 5.0 software (BioRad Laboratories).

### 4.7. Statistical Studies

A two-way ANOVA was used to test the homogeneity of the variances, followed by a Dunnett’s T3 or a Tukey’s statistical test to compare the treated samples with the control (untreated sample) or with each other. The confidence level was set to ≥95%.

## 5. Conclusions

The toxicity and cell activation induced by different Nps in a cell type or tissue can be modified when the Nps are combined. For this reason, the characterization of the synergistic/antagonistic effects induced by Nps with different compositions is very relevant to evaluate the potential beneficial or detrimental effects of the physical mixtures.

The results of this study show how the toxicity of ZnO Nps, which is mainly mediated by the release of Zn^2+^ ions, was modified in combination with other metal oxide Nps (Al_2_O_3_, CeO_2_, TiO_2_, Y_2_O_3_). All Nps, except for the Al_2_O_3_ Nps, had a synergistic effect on the cytotoxicity induced by ZnO Nps in a lymphocytic cell line at low and medium concentrations. However, this trend was inverted at high concentrations (≥400 µg/mL), partially due to the inhibitory effect caused by the Nps on the ZnO Nps solubility.

The combination of Nps also induced changes in the cell signalling mediated by the MAPKs and NFκB compared with the individual Nps and this, in turn, affected the cellular response.

Only Al_2_O_3_ Nps had a protective effect when combined with the ZnO Nps, as confirmed by the viability assays, while CeO_2_ and Y_2_O_3_ Nps induced a synergistic effect on the toxicity and p38 activation. TiO_2_ Nps increased the toxicity induced by ZnO Nps but reduced the phosphorylation of the signalling proteins, thus suggesting that the synergistic toxic effect must be related to other signalling pathways or mechanisms.

In summary, the toxicity and cell activation induced by the combined metal oxide Nps have been described. The combination of different Nps modifies the toxicity of the individual Nps and, hence, the synergistic/antagonistic effect should be considered in the cases of accidental or intended use to achieve a more realistic characterization of the potential beneficial or harmful effects of Nps. This has special relevance for occupational risk assessment. The characterization of the Np mixtures at different doses is also important in order to detect any possible change in the biological effect exerted by the co-exposition.

## Figures and Tables

**Table 1:** Summary of the results observed with the metal oxide nanoparticles and their combination with ZnO nanoparticles in Jurkat cells.

Individual or Combined with ZnO Nps (+ZnO)			Metal Oxide Nanoparticles				
Study	Doses µg/mL		ZnO	Al_2_O_3_	CeO_2_	TiO_2_	Y_2_O_3_
**Cell toxicity**	25–800		--	Non toxic	Toxic at > 400 µg/mL	Toxic at >400 µg/mL	Toxic at > 800 µg/mL
	**+ZnO 60**		Toxic	Decreased	Increased (≤400 µg/mL)	Increased (≤400 µg/mL)	Increased (≤400 µg/mL)
**Zn^2+^ ions** released (µg/mL)	**+ZnO 60**	Np at 50	15.2	15.1	14.6	15.5	14.4
		Np at 800		12.0	5.8	15.3	12.2
**MAPK** (ERK, p38, SAPK/JNK phosphor.)	Np at 5 and 50		--	p-p38 ↑ (50)	p-p38 ↑ (5, 50)	p-ERK ↑ (5, 50)	p-ERK ↑ (5, 50) p-38 ↑ (50)
	**+ZnO 5**		p-p38 ↑↑ p-SAPK/JNK ↑↑	p-p38 ↓ (5) p-SAPK/JNK ↑ (5)	p-p38 ↑↑ p-SAPK/JNK ↑ (5) N.D. (50)	p-ERK ↓↓ p-p38 ↑ (5) ↓↓ (50) p-SAPK/JNK ↓↓	p-ERK N.D. p-p38 ↑↑ p-SAPK/JNK ↑ (5) ↓ (50)
**IκBα** (protein level)	Np at 5 and 50		--	↑ (5) ↓ (50)	↑ (5) ↓ (50)	↑ (5) ↑ (50)	↓ (5,50)
	**+ZnO 5**		↓	≈	≈	↑	≈
**Synergistic effect with ZnO Nps**				**Decreased toxicity and MAPK activation**	**Enhanced toxicity and p-38 activation**	**Enhanced toxicity and decreased MAPK and NFκB activation**	**Enhanced toxicity and p-38 activation.** **Decreased ERK activation**

N.D.: Not detected.

**Table 2:** Summary of metal oxide NPs characterization.

Properties	ZnO	TiO_2_	CeO_2_	Al_2_O_3_	Y_2_O_3_
Average primary particle size, nm (from BET/TEM)	20–100	4–8	4–6	12–21	30–50
Average particle size in water, nm (DLS, Intensity)	530 ± 90	31 ± 1	200 ± 49	312 ± 7	295 ± 50
Zeta-potential (mV) in water	+20.3	+47.0	+33.4	+38.0	+25.1
Phase structure	N.A.	Anatase	N.A.	Gamma	N.A.

N.A.: Not applicable.

## Supplementary Materials

The following are available online at www.mdpi.com/1422-0067/19/1/246/s1.

## Abbreviations

Nps	Nanoparticles
MAPK	Mitogen-activated protein kinases
NFκB	nuclear factor kappa B cell
ERK	extracellular signal-regulated kinase
SAPK/JNK	stress-activated protein kinase/c-Jun amino-terminal kinase
BET/TEM	Brunauer, Emmett and Teller/transmission electron microscopy
DLS	Dynamic light scattering
