# Supplementary material for: Synergistic Effect of Metal Oxide Nanoparticles on Cell Viability and Activation of MAP Kinases and NFκB
Source: Int J Mol Sci. 2018 Jan 15;19(1):246. doi: 10.3390/ijms19010246 (PMC5796194; doi:10.3390/ijms19010246)
Supplement: Supplementary file 1 [file ijms-19-00246-s001.docx]

**Table S1.** Size and Z potential of the physical mixture with ZnO at 60 µg/mL of other metal oxide nanoparticles at two different concentrations (50 and 800 μg/mL). The samples were measured in water by DLS.

| NPs | TiO_2_ | | CeO_2_ | | Al_2_O_3_ | | Y_2_O_3_ | |
| --- | --- | --- | --- | --- | --- | --- | --- | --- |
|  | **+ZnO (60 µg/mL)** | | | | | | | |
| µg/mL | **50** | **800** | **50** | **800** | **50** | **800** | **50** | **800** |
| Size (nm) | 1082±17 | 153±56 | 2944±45 | 1330±31 | 4795±46 | 441±7 | 2781±300 | 649±9 |
| Zeta (mV) | +9 | +27 | +9 | +23 | +13 | +22 | +7 | +31 |

Zeta potential S.D. was very low (0.07-0.56)

**Cell viability determined by an impedance-based assay**

The RTCA xCELLigence RTCA DP from Roche (Basel, Switzerland) was used to perform this viability study with the THP-1 cell line. This equipment measures changes in the impedance induced by cell attachment to and detachment from a gold electrode placed at the bottom of each well and allows cell growth and cell death to be monitored in real time.

The THP-1 cells were seeded at 1 × 10^5^ cells/mL in RPMI supplemented with 10% (v/v) heated-inactivated fetal bovine serum (FBS) (PAA, Austria), 100 U/mL of penicillin/streptomycin and PMA at 64 nM. The cells were incubated for 24 hours to allow them to differentiate into macrophages. After this period, the medium was replaced by fresh medium and the cells were incubated for another 24 hours before adding the Nps. The cell culture medium was removed and replaced by medium with Nps at two different concentrations (100 and 800 µg/mL), alone or mixed with ZnO Nps at 60 µg/mL. Cell viability was monitored for another 48 hours after adding the Nps.


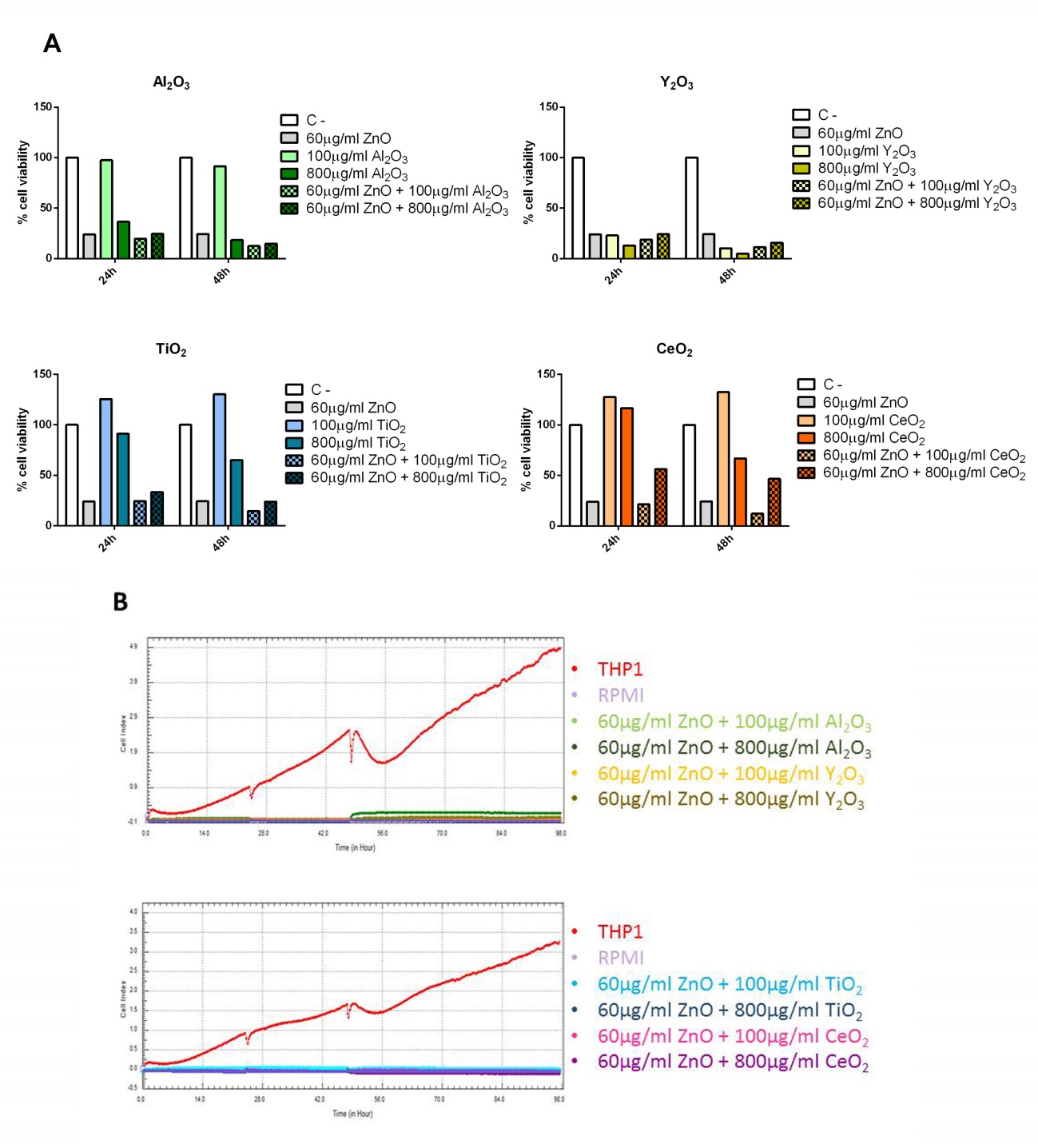


**Figure S1.** **A.** Cell viability of macrophage-differentiated THP-1 cells after 24 hours of incubation with 60 µg/mL ZnO Nps alone or combined with Al_2_O_3_, CeO_2_, TiO_2_ or Y_2_O_3_ Nps at 100 or 800 µg/mL determined by the RTCA xCELLigence system. The viability of the cells was monitored for 48 hours and the individual Nps were also tested. **B.** Nps alone in cell culture medium were also included as negative control and non-interaction was detected with the electrodes.


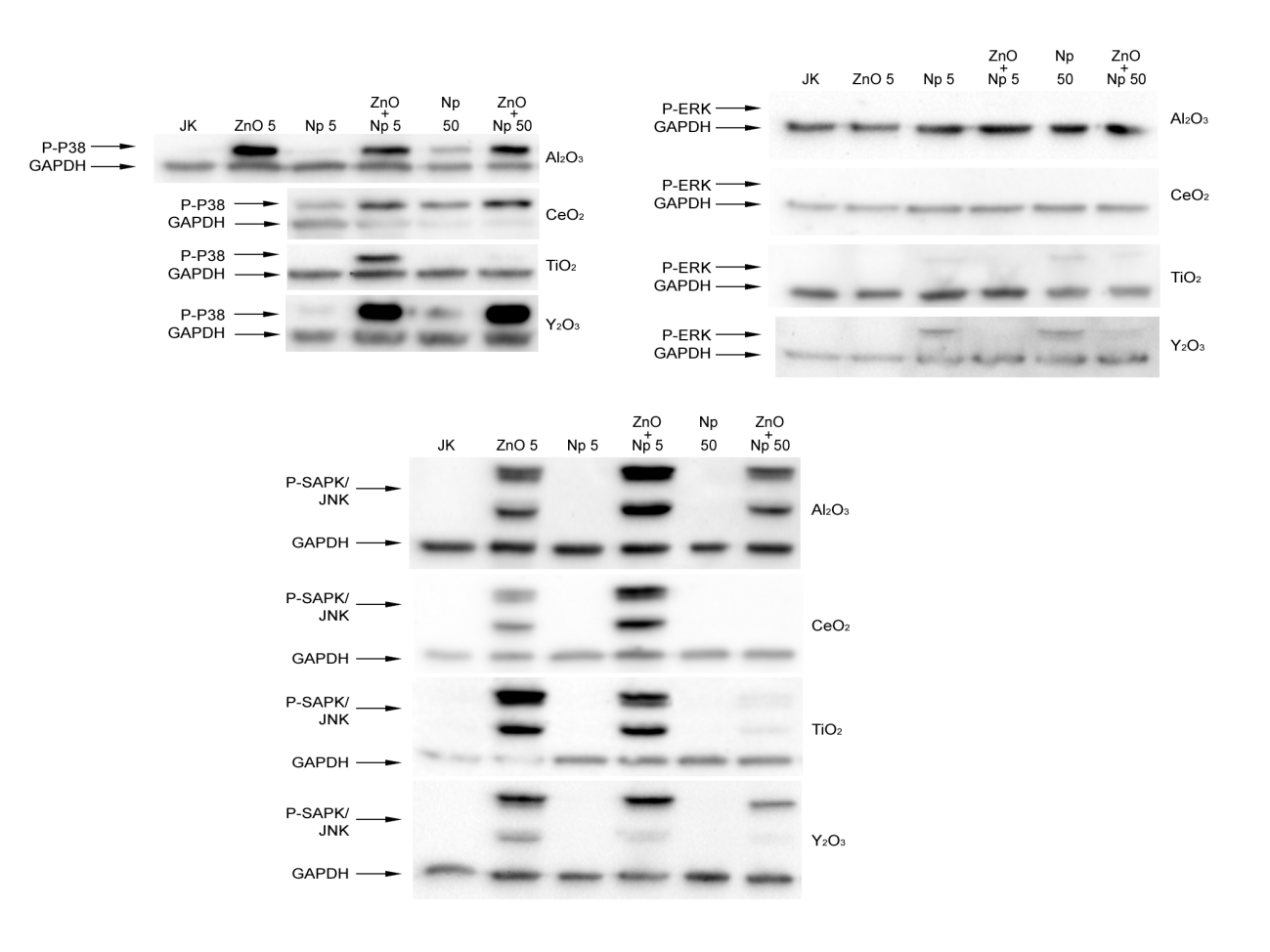


**Figure S2.** Expression of p-p38, p-ERK, p-SAPK/JNK in Jurkat cells. Western blots show the expression of p-p38, p-ERK and p-SAPK/JNK in cells treated with Al_2_O_3_, CeO_2_, TiO_2_ and Y_2_O_3_ at different concentrations (5 and 50 µg/mL) and combined with 5 µg/mL ZnO. These results shown belong to a representative experiment (n = 3).
